# A simple proteinuria-based risk score predicts contrast-associated acute kidney injury after percutaneous coronary intervention

**DOI:** 10.1038/s41598-022-16690-6

**Published:** 2022-07-19

**Authors:** Wakaya Fujiwara, Hideki Ishii, Yoshihiro Sobue, Shinya Shimizu, Tomoya Ishiguro, Ryo Yamada, Sayano Ueda, Hideto Nishimura, Yudai Niwa, Akane Miyazaki, Wataru Miyagi, Shuhei Takahara, Hiroyuki Naruse, Junichi Ishii, Ken Kiyono, Eiichi Watanabe, Hideo Izawa

**Affiliations:** 1 Division of Cardiology, Department of Internal Medicine, Fujita Health University Bantane Hospital, 3-6-10 Otobashi, Nakagawa, Nagoya, 454-8509 Japan; 2 Department of Cardiology, Fujita Health University School of Medicine, Toyoake, Japan; 3 Department of Cardiovascular Medicine, Graduate School of Medicine, Gunma University, Maebashi, Japan; 4 Division of Bioengineering, Graduate School of Engineering Science, Osaka University, Suita, Japan

**Keywords:** Predictive markers, Interventional cardiology

## Abstract

Contrast-associated acute kidney injury (CA-AKI) is a complication of percutaneous coronary intervention (PCI). Because proteinuria is a sentinel marker of renal dysfunction, we assessed its role in predicting CA-AKI in patients undergoing PCI. A total of 1,254 patients undergoing PCI were randomly assigned to a derivation (*n* = 840) and validation (*n* = 414) dataset. We identified the independent predictors of CA-AKI where CA-AKI was defined by the new criteria issued in 2020, by a multivariate logistic regression in the derivation dataset. We created a risk score from the remaining predictors. The discrimination and calibration of the risk score in the validation dataset were assessed by the area under the receiver-operating characteristic curves (AUC) and Hosmer–Lemeshow test, respectively. A total of 64 (5.1%) patients developed CA-AKI. The 3 variables of the risk score were emergency procedures, serum creatinine, and proteinuria, which were assigned 1 point each based on the correlation coefficient. The risk score demonstrated a good discriminative power (AUC 0.789, 95% CI 0.766–0.912) and significant calibration. It was strongly associated with the onset of CA-AKI (Cochran-Armitage test, *p* < 0.0001). Our risk score that included proteinuria was simple to obtain and calculate, and may be useful in assessing the CA-AKI risk before PCI.

## Introduction

Contrast-associated acute kidney injury (CA-AKI) is a serious complication commonly associated with percutaneous coronary intervention (PCI)^1–5^. CA-AKI can lead to extended hospital stays, unplanned hemodialysis, and an increased risk of death^4–6^. The exact mechanism of CA-AKI has not been elucidated but CA-AKI involves a complex interaction of several mechanisms including vasoconstriction, renal medulla ischemia, tubular damage due to contrast, and cholesterol embolism syndrome^4,5^. Because no specific treatment exists, the most effective strategy remains to be prevention with prophylactic intravenous normal saline^7^.

To date, many risk models have been published to predict the factors that predispose PCI patients to CA-AKI and have proven useful in clinical practice^8–11^. A clinical factor common to most risk scores is renal dysfunction, while risk factors such as old age, diabetes, hypotension, heart failure, the contrast volume, and emergency treatment are also recognized^5^. Proteinuria is well known to be a significant risk factor for adverse cardiovascular events across a range of populations^12,13^. Proteinuria is a sign of glomerular damage even in patients with a seemingly normal renal function, and could indicate a higher vulnerability to CA-AKI. However, few studies have examined whether proteinuria has a predictive role in the development of CA-AKI in patients undergoing PCI^14^.

Formerly, CA-AKI had been defined as an increase of > 0.5 mg/dL or > 25% in the baseline serum creatinine (SCr) level, within 48–72 h after exposure to contrast media, excluding other causes of renal function impairment^4^. In 2020, however, the American College of Radiology and the National Kidney Foundation defined new diagnostic criteria for CA-AKI: > 0.3 mg/dL or a > 50% increase in the baseline SCr value within 48 h after contrast exposure^15^. Currently, few studies have compared the incidence of CA-AKI or have constructed a predictive model for CA-AKI based on its new definition^16^. The purpose of this study, therefore, was to examine the incidence of CA-AKI after PCI based on its new definition, assess the role of proteinuria, and construct a risk score to assess the CA-AKI risk.

## Results

### Patients

During the recruitment period, 1,281 consecutive patients were assessed for enrollment eligibility. We excluded 27 patients based on predetermined criteria. In the elective PCI patients, urinary protein levels were measured 13 (interquartile range 2 – 25) days prior to the PCI (*n* = 1019), and the urinary protein level was measured in all those patients prior to preprocedural hydration. The longest time from the urinalysis to the PCI was 89 days (n = 1). Of the 1,254 patients, 64 (5.0%) developed CA-AKI. Table 1 shows the baseline clinical features of the patients with and without development of CA-AKI. The mean age of all patients was 70.1 ± 11.0 years and 72.2% were male. Patients who developed CA-AKI were older, had a lower body mass index, and more commonly had an emergency PCI, hypertension, heart failure, chronic kidney disease (CKD), and proteinuria. Patients with CA-AKI also had higher SCr and N-terminal pro-brain natriuretic peptide values and a lower estimated glomerular filtration ratio (eGFR) and left ventricular ejection fraction (LVEF). The mean volume of the contrast was 175.9 ± 64.5 mL and did not significantly differ between the patients with and without CA-AKI. Patients with CA-AKI were more likely to receive either intra-aortic balloon pumping or extracorporeal membrane oxygenation. The incidence of CA-AKI was significantly higher under the new diagnostic criteria (n = 64, 5.0%, 95% CI 4.0 – 6.5%, *p* < 0.001) than under the old criteria (n = 24, 1.9%, 95% CI 1.2 – 2.8%). The proportion of patients with an impaired renal function as assessed by the SCr and eGFR values and the incidence of CA-AKI increased in proportion to the severity of the proteinuria (Supplementary Table 1).

**Table 1:** Baseline characteristics.

	Contrast-associated acute kidney injury (-) (*n* = 1190)	Contrast-associated acute kidney injury ( +) (*n* = 64)	*P*-value
Age, years	69.9 ± 11.1	73.7 ± 9.4	< 0.01
Male, n (%)	867 (72.9)	40 (62.5)	0.08
BMI, kg/m^2^	24.0 ± 4.2	22.4 ± 4.2	< 0.01
SBP, mmHg	134 ± 21	131 ± 24	0.35
DBP, mmHg	71 ± 14	69 ± 14	0.34
Emergency procedure, n (%)	207 (17.4)	28 (43.8)	< 0.01
STEMI	127 (10.7)	18 (28.1)	< 0.01
NSTEMI	80 (6.7)	10 (15.6)	0.02
**Comorbidities, n (%)**			
Hypertension	943 (79.2)	59 (92.2)	< 0.01
Diabetes mellitus	478 (40.2)	33 (51.6)	0.07
Heart failure	241 (20.3)	36 (56.3)	< 0.01
Dyslipidemia	825 (69.3)	34 (53.1)	< 0.01
Prior myocardial infarction	373 (31.3)	14 (21.9)	0.10
CKD	501 (42.1)	54 (84.4)	< 0.01
Smoking	314 (26.4)	18 (28.1)	0.76
**Laboratory data**			
Hematocrit, %	39.3 ± 5.1	35.9 ± 6.1	0.78
LDL-C, mg/dL	106.4 ± 35.5	109.2 ± 38.3	0.55
HDL-C, mg/dL	50.3 ± 13.9	50.5 ± 21.0	0.91
Triglyceride, mg/dL	150.7 ± 95.7	145.4 ± 79.0	0.66
FBS, mg/dL	144.9 ± 61.9	156.3 ± 79.1	0.18
HbA1c, %	6.7 ± 3.2	6.8 ± 1.5	0.85
SCr, mg/dL	0.9 ± 0.3	1.4 ± 0.6	< 0.01
eGFR, ml/min/1.73m^2^	63.0 ± 19.1	43.7 ± 17.0	< 0.01
NT-proBNP, pg/mL	1000 ± 3260	5898 ± 12,432	< 0.01
Proteinuria, n (%)	269 (22.6)	39 (60.9)	< 0.01
LVEF, %	61.4 ± 12.1	52.5 ± 14.4	< 0.01
Contrast media volume, ml	175.4 ± 63.1	188.1 ± 66.1	0.79
IABP/ECMO, n (%)	24 (2.0)	7 (10.9)	< 0.01
**Medications**, n (%)			
Aspirin	993 (83.4)	40 (62.5)	< 0.01
ACEI	142 (11.9)	13 (20.3)	0.06
ARB	617 (51.8)	32 (50.0)	0.77
β-blocker	403 (33.9)	21 (32.8)	0.86
Calcium channel blocker	544 (46.6)	38 (59.4)	0.03
Oral hypoglycemic agents (excluding metformin)	416 (35.0)	29 (45.3)	0.09
Metformin	113 (9.5)	5 (7.8)	0.64
Insulin	62 (5.2)	7 (10.9)	0.08
Statin	803 (67.5)	26 (40.6)	< 0.01
Loop diuretics	216 (18.2)	26 (40.6)	< 0.01
MRA	67 (5.6)	5 (7.8)	0.49

ACEI, angiotensin-converting enzyme inhibitor; ARB, angiotensin II receptor blocker; BMI, body mass index; CKD, chronic kidney disease; DBP, diastolic blood pressure; ECMO, extracorporeal membrane oxygenation; eGFR, estimated glomerular filtration rate; FBS, fasting blood sugar; HbA1c, hemoglobin A1c; HDL-C, high-density lipoprotein cholesterol; IABP, intra-aortic balloon pumping; LDL-C, low-density lipoprotein cholesterol; LVEF, left ventricular ejection fraction; MRA, mineralocorticoid receptor inhibitor; NSTEMI, non-ST elevation myocardial infarction; NT-proBNP, N-terminal pro-brain natriuretic peptide; Proteinuria, >  = ( ±) by dipstick; SBP, systolic blood pressure; SCr, serum creatinine; STEMI, ST elevation myocardial infarction. Data are presented as the number, frequency, and mean ± SD.

### Risk model development

The baseline clinical features of the patients in the derivation dataset and validation dataset are presented in Supplementary Table 2. CA-AKI occurred in 39 (4.6%) patients from the derivation dataset. All variables listed in Table 1 were used in the univariate analysis. The significant univariate variables associated with the occurrence of CA-AKI in the derivation dataset are shown in Supplementary Table 3. The independent predictors of CA-AKI in the multivariate model are shown in Table 2. There were no significant multicollinearities among the 4 factors. The AUC of this model was 0.866 (95%CI 0.802–0.930), and the calibration curve showed that the predictive probability was significantly associated with the actual probability of CA-AKI (Hosmer–Lemeshow test, χ^2^ = 6.14, *P* = 0.63) (Supplementary Fig. 1A).

**Table 2:** A multivariate logistic regression analysis.

Variables	Regression coefficient	SE	OR (95% CI)	*P*-value
SCr, per 1 mg/dL	1.396	0.377	4.04 (1.93–8.47)	< 0.001
Emergent procedure	1.218	0.383	3.38 (1.59–7.17)	< 0.001
LVEF, per 1%	− 0.038	0.013	0.96 (0.94–0.99)	< 0.001
Proteinuria	1.413	0.394	4.11 (1.90–8.91)	< 0.001
Intercept	− 3.379			

The abbreviations are presented in Table 1. *SE* standard error, *OR* odds ratio, *CI* confidence interval. Multicollinearity diagnostics (variance inflation factor): SCr = 1.092, emergent procedure = 1.029, LVEF = 1.036, urine protein = 1.095.

### Risk score development

**Figure 1:**
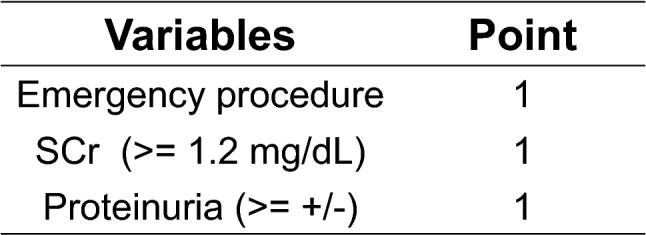
Predictive score for contrast-associated acute kidney injury (CA-AKI). The regression coefficients estimated from the logistic model were used to develop the score. The cutoff value of the SCr was calculated according to the Youden index. Akaike information criterion = 228.26. SCr: serum creatinine.

**Figure 2:**
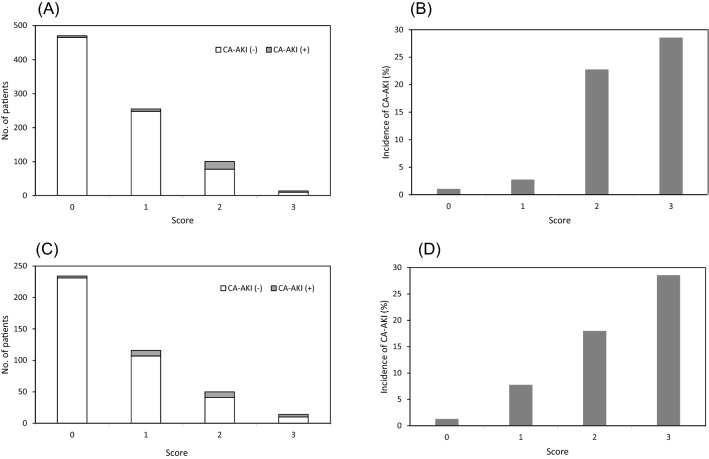
Actual number and incidence of CA-AKI as a function of the risk score. (**A**) Number of patients with CA-AKI and (**B**) incidence of CA-AKI in the derivation dataset. (**C**) Number of patients with CA-AKI and (**D**) incidence of CA-AKI in the validation dataset. An increasing risk of CA-AKI with an increasing risk score is evident. CA-AKI: contrast-associated acute kidney injury.

A weighted score of 1 was assigned to 3 of the 4 independent factors, an emergency procedure, the SCr, and proteinuria, based on the regression coefficient. The LVEF was not included in the risk score because its correlation coefficient was 2 orders of magnitude smaller than the other three risk factors. (Fig. 1). Therefore, the risk scores in the patients ranged between 0 and 3 points. Increasing the score values increased the incidence of CA-AKI (Cochran-Armitage trend test, *p* < 0.0001) (Fig. 2A,B).

### Risk score validation

**Figure 3:**
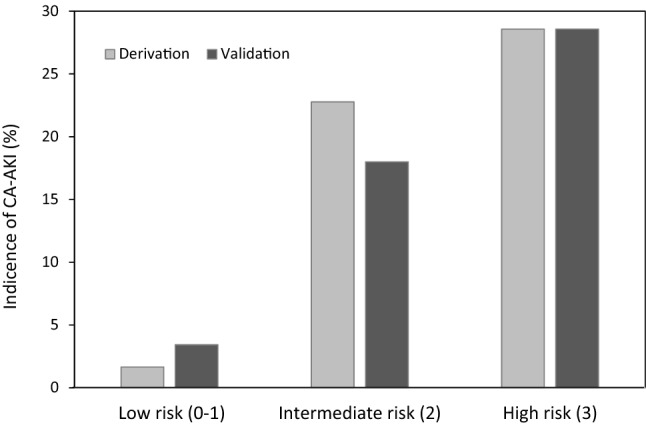
Incidence of CA-AKI according to the three risk groups. The incidence of CA-AKI in the derivation and validation dataset stratified into 3 groups (low risk, intermediate risk, and high risk) is shown.

CA-AKI occurred in 25 (6.0%) of 414 patients from the validation set. Our CA-AKI prediction score demonstrated a good discriminative power (AUC 0.789, 95%CI 0.710 – 0.868) and calibration (Hosmer–Lemeshow test, χ^2^ = 10.6, *p* = 0.22) (Supplementary Fig. 1B). Also, the bootstrapping internal validation yielded an average AUC of 0.781 (bias-corrected 95% CI, 0.651–0.886). There were again significant trends across the increasing score values for the prediction of CA-AKI (Cochran-Armitage trend test, *p* < 0.0001) (Fig. 2C,D). Based on the obtained frequencies of the CA-AKI in relation to the risk score, the patients were further categorized into three groups: low risk (0 – 1 point), intermediate risk (2 points), and high risk (3 points). Figure 3 shows the incidence of CA-AKI for the three risk score groups separately for the derivation and validation cohort.

### Risk score and hemodialysis after the PCI

**Figure 4:**
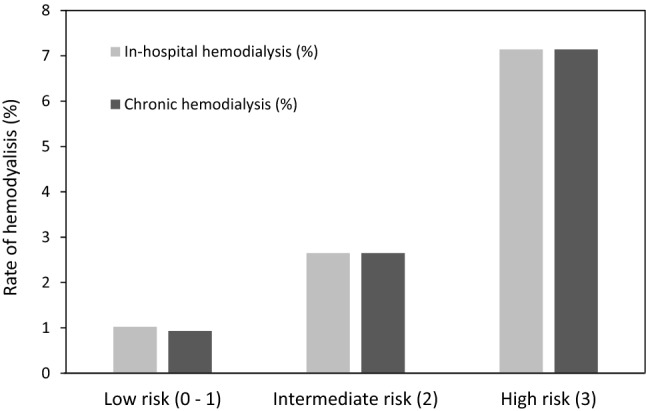
Incidence of hemodialysis according to the three risk groups. The incidence of in-hospital and chronic hemodialysis in the total dataset stratified into 3 groups (low risk, intermediate risk, and high risk) is shown.

The ability of the risk score to predict the rates of post-PCI hemodialysis was further evaluated in the total dataset. Sixteen patients required transient hemodialysis; their in-hospital mortality was 25%. Three patients were discharged on chronic hemodialysis and an additional 13 patients came to require chronic hemodialysis after discharge. Significant increases in the rates of transient and chronic hemodialysis were observed with an increasing risk score (Cochran-Armitage trend test, *p* < 0.0001) (Fig. 4).

### Performance compared to the risk scores formulated by others

We compared the discrimination performance of our risk score with that of Mehran et al.^8^, Ranucci et al.^10^, Ando et al.^17^, Liu et al.^18^, and Inohara et al.^9^ using our validation dataset (Table 3). The predictive performance of our risk score was superior to the scores published by Ranucci, Ando, and Liu, and was comparable to that published by Mehran and Inohara.

**Table 3:** Comparison of the risk score performance.

	AUC	95% CI	*P*-value
Our score	0.785	0.700–0.871	
Mehran	0.769	0.675–0.863	0.745
Ranucci	0.618	0.521–0.716	0.018
Ando	0.652	0.583–0.722	0.008
Liu	0.667	0.644–0.691	0.008
Inohara	0.803	0.721–0.886	0.620

*AUC* area under the curve, *CI* confidence interval.

### Performance of the risk prediction in patients with and without cardiogenic shock

Cardiogenic shock influences the renal function and amount of proteinuria. In this study, we included 31 (2.4%) cardiogenic shock patients that used an intra-aortic balloon pumping and/or extracorporeal membrane oxygenation system (Table 1). We compared the performance of the risk prediction in the entire dataset (*n* = 1254) and dataset excluding cardiogenic shock (*n* = 1223). The AUC of the entire cohort was 0.810 (95%CI 0.756–0.865), and the corresponding value for the cohort excluding cardiogenic shock was 0.803 (95%CI 0.744–0.863) (*p* = 0.63). The predictive performance of our risk score did not change even after including cardiogenic shock patients.

## Discussion

### Major findings

In this study, we found that the incidence of CA-AKI was greater under the new 2020 diagnostic criteria. Our risk model, which used only three risk factors (emergency procedures, SCr, and proteinuria) showed a good discrimination and significant calibration in the validation dataset. An increasing risk score was strongly associated with the development of CA-AKI as well as in-hospital and post-discharge hemodialysis. Our risk model may serve as an easy to apply guide for evaluating the individual patient risk for the development of CA-AKI after the PCI.

CA-AKI accounts for roughly 10% of hospital-acquired renal failure, but the incidence of CA-AKI depends on the definition used. Formerly, CA-AKI had been defined as an increase of > 0.5 mg/dL or > 25% in the baseline SCr within 48–72 h after exposure to contrast media^4^. The newer guideline issued in 2020 lowered the threshold for an absolute increase in the SCr, but increased the threshold for a relative increase, as well as limiting the time period to 48 h, making it unclear whether the new criteria would lead to an increase or decrease in the incidence of CA-AKI by definition. In 2020, the American College of Radiology and the National Kidney Foundation issued a new definition of an absolute (0.3 mg/dL within 48 h after contrast exposure) or relative (> 50%) increase in the baseline SCr, and this can be a standard reference for defining both the onset and severity of CA-AKI^15^. In our study, the incidence of CA-AKI increased significantly from that under the old definition, 1.9%, to that under the new definition, 5.0%. In a recent report, the optimal definition of CA-AKI in predicting adverse cardiovascular outcomes and post-PCI mortality was compared according to four different definitions: an absolute elevation in the SCr of ≥ 0.3 mg/dL or ≥ 0.5 mg/dL at 48 h post PCI or a relative elevation of ≥ 25% or ≥ 50%^19^. They found that an absolute elevation of ≥ 0.3 mg/dL in the SCr 48 h post PCI predicted the outcomes most accurately. The new definition may have increased the sensitivity of the CA-AKI diagnosis and led to a rise in the cardiologists' awareness of CA-AKI.

A majority of the prior studies reported that mild-to-moderate renal dysfunction was a risk factor for CA-AKI^8–10,18^. In agreement, the SCr was an independent predictor of CA-AKI in our study. An emergency procedure was also associated with CA-AKI, consistent with the previous reports, probably due to vasoconstriction or a reduced renal blood flow associated with a sudden hemodynamic deterioration caused by acute coronary syndrome. In our study, the contrast dose was not independently associated with an increased risk of CA-AKI in the logistic analysis. That was probably because the operators performed the PCI with a low amount of contrast given the severity of the renal function shown in Supplementary Table 2. Our observation that the contrast media was not an independent predictor of CA-AKI in the patients undergoing a PCI was in agreement with the recent literature^9,10,16^.

The main strength of our risk model was that it stratified the patients at risk of CA-AKI well using only 3 pre-procedural factors. We could explore the useful risk score using inexpensive and routinely collected measurements in the preprocedural setting. We have shown that our risk score achieved a similar predictive value as compared to the Mehran’s score^8^ and Inohara’s score^9^. Mehran’s risk score, which has been widely used from 2004, includes 8 factors: 6 pre-procedural factors (age > 75 years, congestive heart failure, anemia, diabetes, hypotension, and eGFR) and 2 procedural factors (intra-aortic balloon pump and the contrast media volume). Inohara’s 7 risk score factors are all pre-procedural: age, heart failure, diabetes, previous PCI, hypertension, SCr, and acute coronary syndrome. A simpler, intuitive, and easily obtainable risk score may be useful given the high morbidity of CA-AKI and the importance of early detection. Recently, Allen et al. examined the discrimination and calibration of the risk prediction models for CA-AKI accompanying cardiac catheterization in a meta-analysis^5^. They found that CA-AKI prediction models had a good discrimination (C-statistic 0.78) but high heterogeneity (I^2^ statistic = 95.8%, Cochran Q-statistic *P* < 0.001), partly due to the differences in the CA-AKI definitions used in each study. They further reported that models that included postprocedure in addition to preprocedure variables did not exhibit significantly higher c-statistics than the models that used only preprocedure variables. A preprocedural risk stratification provides cardiologists with an opportunity to better understand the risks of CA-AKI before embarking on a PCI, providing for kidney protective strategies such as preprocedural hydration and minimization of the contrast volume.

In this study we showed that proteinuria was independently predictive of CA-AKI in patients undergoing a PCI. Our results were supported by a previous study, which reported an incremental value in the proteinuria for predicting CA-AKI after cardiac catheterization^14^. While proteinuria is thought to be a manifestation of glomerular damage, recent experimental data has shown that albumin, which accounts for most of the urinary protein content, upregulates the expression of pro-inflammatory and profibrotic mediators in cultured renal tubular cells^20^. These results suggest that ultrafiltered albumin secreted from renal tubular cells damages the renal function via complement activation or an inflammatory process leading to interstitial fibrosis and tubular damage. Patients with proteinuria may have an impaired physiological adaptability and be less tolerant of renal hemodynamic changes and nephrotoxic injuries such as from contrast media.

Proteinuria is known to be associated with various types of renal diseases including glomerulonephritis, urolithiasis, infection and malignancy, or other causes including hypertensive crisis, cardiogenic shock, strenuous exercise, and a cold or febrile condition. While we could not always exclude those conditions, our risk score incorporating proteinuria had a good performance for predicting CA-AKI. Further study is needed to determine whether contrast media affects the amount of proteinuria in patients undergoing PCI.

### Limitations

This was a retrospective and single-center study that excluded patients undergoing emergency cardiac surgery and patients who died within 2 days after the procedure. Such patients may have a high prevalence of renal dysfunction or various comorbidities. Thus, we likely underestimated the incidence of CA-AKI. However, the number of patients excluded for any reason was 27 out of 1,281. Secondly, we evaluated our model using an internal validation dataset. Thirdly, we determined the severity of the proteinuria using the dipstick method because it allows for an easy and rapid diagnosis. The dipstick method, however, evaluates gross albuminemia (urinary albumin excretion > 300 mg/day) rather than microalbuminuria (30–300 mg/day). In a future study, we need to test the effect of microalbuminemia in assessing the risk of CA-AKI. In addition, the dipstick test can give false negative results^21^.

## Conclusion

The incidence of CA-AKI after PCI was higher under the new 2020 diagnostic criteria for CA-AKI. We were able to assess the risk of post-PCI CA-AKI and renal damage requiring chronic hemodialysis by assigning 1 point to each of 3 simple factors, any level of proteinuria, SCr > 1.2 mg/dl, and whether the PCI was emergent or not.

## Methods

### Ethics statement

The protocol for the study was approved by the Ethical Review Board for Epidemiological and Clinical Studies of the Fujita Health University School of Medicine (Approval No.: HM17-104) in compliance with the Declaration of Helsinki. All participants provided informed written consent.

### Study population

We retrospectively examined the records from 1,281 consecutive patients who had undergone an elective or emergency PCI between January 2010 and December 2019 at our hospital. Patients aged ≥ 18 years of either gender were considered eligible. Patients were excluded for any of the following reasons: end-stage renal disease requiring dialysis, uncontrolled diabetes, New York Heart Association (NYHA) class IV heart failure, and administration of iodinated contrast media within 72 h prior to the PCI. We also excluded patients who did not receive a urinalysis before the PCI, patients who transitioned to emergency cardiac surgery, and those who died within 2 days of the PCI. We used low-osmolar non-ionic contrast media, either iomeprol (Iomeron 350, Bracco Imaging) or 54 iohexol (Omnipaque 350, GE Healthcare). In patients undergoing an elective PCI whose eGFR was < 45 mL/min/1.73m^2^, we administered 1 mL/kg/hour of normal saline 6—12 h before and 6 – 12 h after the PCI. In patients who underwent an emergency PCI, short-term fluid replacement was not implemented, but some received normal saline 6 – 12 h after the PCI at the discretion of the operator. All procedures were performed using standard cardiac catheterization techniques via the femoral or radial approach, and mechanical circulatory support including intra-aortic balloon pumping or extracorporeal membrane oxygenation was used in case of cardiogenic shock. We have stated the sample size estimation in the Supplementary file.

### Study protocol

The baseline data included the clinical, demographic, procedural, and angiographic characteristics, medications used before the procedure, and in-hospital outcomes. In the elective PCI patients, the baseline laboratory test results were defined as the last reported values within 3 months prior to the procedure. Among the patients who had multiple assessments of the SCr level in the 3 months before the procedure, the value closest to the time of the procedure was considered the baseline value. In patients undergoing an emergent PCI, a 12-lead ECG, echocardiography, and blood samples were taken in the emergency room. Urine samples were taken from their urine bags. For the proteinuria analysis, we used a dipstick (Uropaper III dipsticks [Eiken Chemical Co., Ltd., Tokyo, Japan]) to perform a semi-quantitative assessment of the proteinuria via a urine chemistry analyzer (us-2200, Eiken Chemical Co., Ltd., Tokyo, Japan). The results were reported in a semi-quantitative manner, namely, (–), ( ±), (1 +), (2 +), or (3 +). The (–) was defined as a negative proteinuria, and the rest were defined as a positive proteinuria. The SCr was measured on days 1, 2, and 3 after the PCI. The peak SCr was defined as the highest value of the SCr within 48 h following the procedure. In case of an emergency procedure, the blood pressure was measured in the emergency department. Wall motion abnormalities and the LVEF were assessed in the emergency department.

### Definitions and endpoints

Diabetes mellitus (DM) was diagnosed if the patient was taking a hypoglycemic drug or had an HbA1c level ≥ 6.5% (48 mmol/mol). Significant heart failure was defined as NYHA functional class ≥ 2, Killip class ≥ 1, or pulmonary edema. Hypertension was diagnosed when the patient's systolic or diastolic blood pressure was > 140 mmHg or > 90 mmHg, respectively, or if they were on antihypertensive medications. CKD was diagnosed if the patient had an eGFR < 60 ml/min per 1.73 m^2^. The primary endpoint was CA-AKI, which was defined according to the 2020 diagnostic criteria, to reiterate, a > 0.3 mg/dL increase in the SCr level from baseline or a ≥ 50% increase from baseline within 48 h of the contrast-medium exposure. The secondary endpoints were a transient need for hemodialysis during the hospitalization for the PCI and the need for chronic hemodialysis after discharge from the hospital.

### Statistical analysis

The continuous variables are presented as mean ± standard deviation (SD) values and were compared using a Student’s t-test. The categorical data were presented as the number and frequency evaluated using a Pearson’s Chi-squared test. A two-tailed p-value of < 0.05 was considered significant. All statistical data were analyzed using JMP version 15.1.0 (SAS Institute, Cary, NC, USA), R project (Version 4.0.5), or EZR software^22^.

### Risk score construction

Eligible patients from the entire database were randomized in a 2:1 ratio to create a derivation (*n* = 840) and validation (*n* = 414) dataset. The derivation dataset was used for identifying univariate associations between the baseline clinical and procedural characteristics and CA-AKI. Variables with a *P* < 0.1 in the univariate logistic analysis were available for a further multivariate regression. A stepwise selection method was used to construct the best model on the basis of the Akaike information criterion with an acceptable low multicollinearity (variance inflation factor) of the selected variables. The independent variables in the final models were assigned a weighted integer coefficient value. The risk score represented the sum of the integer coefficients. For scoring purposes, the continuous variable SCr was dichotomized using the best cutoff value according to the Youden index. The sensitivity and specificity of the cutoff value of SCr were 83% and 65%, respectively. After finding the range of the risk score values ranging from 0 to 3, the score was categorized into levels of low, intermediate, and high, to enhance the clinical utility. The bootstrap method with 1000 replications was used to perform an internal cross-validation of the risk score model. The discrimination was assessed using the area under the receiver-operating characteristic (ROC) curve, and the calibration was assessed using the Hosmer–Lemeshow goodness of fit test. The area under the ROC curves were compared using the DeLong test. The performance of the risk score was tested by the Cochran-Armitage trend test. Finally, the significance of the risk score for the rates of in-hospital dialysis and chronic dialysis was estimated.

## IRB information

The Fujita Health University Ethical Review Board for Epidemiological and Clinical Studies approved this study (Approval No. HM17-104).

## Supplementary Information

Supplementary Information 1.

## Data Availability

The data that support the findings of this study are available from the corresponding author upon reasonable request.
