# Supplementary material for: A simple proteinuria-based risk score predicts contrast-associated acute kidney injury after percutaneous coronary intervention
Source: Sci Rep. 2022 Jul 19;12:12331. doi: 10.1038/s41598-022-16690-6 (PMC9296582; doi:10.1038/s41598-022-16690-6)
Supplement: Supplementary file 1 — Supplementary Information 1. [file 41598_2022_16690_MOESM1_ESM.docx]

**Supplementary Table 1. Baseline characteristics grouped by proteinuria**

|  | **Negative**  **Proteinuria**  **(n = 946)** | **Low proteinuria**  **(+/-)**  **(n = 95)** | **High proteinuria**  **(>=1+)**  **(n = 213)** | **p-value** |
| --- | --- | --- | --- | --- |
| Age (years) | 70 ± 11 | 73 ± 11 | 71 ± 10 | 0.01 |
| Male, n (%) | 687 (72.6) | 66 (69.5) | 153(71.8) | 0.79 |
| BMI (kg/m^2^) | 23.9 ± 4.2 | 22.2 ± 3.9 | 24.4 ± 4.4 | 0.03 |
| SBP (mmHg) | 133 ± 21 | 131 ± 24 | 137 ± 20 | 0.03 |
| DBP (mmHg) | 71 ± 14 | 69 ± 13 | 72 ± 13 | 0.25 |
| Emergency procedure, n (%) | 169 (17.9) | 27 (28.4) | 39 (18.3) | 0.04 |
| STEMI, n (%) | 108 (64.7) | 17 (63.0) | 20 (51.3) | 0.09 |
| NSTEMI, n (%) | 61 (36.5) | 10 (37.0) | 19 (48.7) | 0.20 |
| Comorbidities, n (%) |  |  |  |  |
| Hypertension | 738 (78.0) | 83 (87.4) | 181 (85.0) | 0.01 |
| Diabetes mellitus | 322 (34.0) | 52 (54.7) | 137 (64.3) | <0.01 |
| Heart failure | 164 (17.3) | 35 (36.8) | 78 (36.6) | <0.01 |
| Dyslipidemia | 656 (69.3) | 66 (69.5) | 137 (64.3) | 0.35 |
| Old myocardial infarction | 301 (31.8) | 18 (18.9) | 68 (31.9) | 0.03 |
| CKD | 321 (33.9) | 67 (70.5) | 167 (78.4) | <0.01 |
| Smoking | 248 (26.2) | 25 (26.3) | 59 (27.7) | 0.91 |
| **Laboratory data** |  |  |  |  |
| Hematocrit (%) | 39.7 ± 5.1 | 37.9 ± 5.9 | 38.1 ± 5.3 | 0.74 |
| LDL-C (mg/dL) | 106.5 ± 34.7 | 102.8 ± 39.4 | 108.4 ± 37.8 | 0.44 |
| HDL-C (mg/dL) | 51.2 ± 13.9 | 46.5 ± 14.3 | 48.3 ± 16.0 | <0.01 |
| Triglyceride (mg/dL) | 149.0 ± 96.9 | 144.1 ± 92.3 | 159.0 ± 86.8 | 0.31 |
| FBS (mg/dL) | 138.6 ± 54.9 | 168.7 ± 71.2 | 161.9 ± 95.1 | <0.01 |
| HbA1c (%) | 6.6 ± 3.5 | 7.0 ± 1.8 | 7.1 ± 1.5 | 0.06 |
| SCr (mg/dL) | 0.9 ± 0.2 | 1.1 ± 0.4 | 1.2 ± 0.7 | <0.01 |
| eGFR (ml/min/1.73m^2^) | 66.0 ± 17.8 | 52.2 ± 20.5 | 48.6 ± 18.5 | <0.01 |
| NT-proBNP (pg/mL) | 774 ± 2026 | 2737 ± 6240 | 2703 ± 8529 | <0.01 |
| LVEF (%) | 61.9 ± 11.8 | 57.5 ± 14.2 | 58.1 ± 13.4 | <0.01 |
| Contrast media volume (ml) | 177.9 ± 63.1 | 169.2 ± 62.9 | 171.0 ± 63.9 | 0.19 |
| CA-AKI, n (%) | 23 (2.4) | 12 (12.6) | 29 (13.6) | <0.01 |
| IABP/ECMO, n (%) | 18 (1.9) | 5 (5.3) | 8 (3.8) | 0.06 |
| **Medications**, n (%) |  |  |  |  |
| Aspirin | 791 (83.6) | 73 (76.8) | 169 (79.3) | 0.11 |
| ACEI | 108 (11.4) | 15 (15.8) | 32 (15.0) | 0.20 |
| ARB | 468 (49.5) | 56 (58.9) | 125 (58.7) | 0.02 |
| β-blocker | 326 (34.5) | 32 (33.7) | 66 (31.0) | 0.63 |
| Calcium channel blocker | 411 (43.4) | 44 (46.3) | 127 (59.6) | <0.01 |
| Oral hypoglycemic agents* | 286 (30.2) | 42 (44.2) | 117 (54.9) | <0.01 |
| Metformin | 76 (8.0) | 13 (13.7) | 29 (13.6) | 0.01 |
| Insulin | 29 (3.1) | 15 (15.8) | 25 (11.7) | <0.01 |
| Statin | 645 (68.2) | 64 (67.4) | 120 (56.3) | <0.01 |
| Loop diuretics | 134 (14.2) | 34 (35.8) | 74 (34.7) | <0.01 |
| MRA | 42 (4.4) | 5 (5.3) | 25 (11.7) | <0.01 |

Patients were classified into negative proteinuria ([–] by dipstick), low proteinuria ([±]), and high proteinuria ([1+] - [3+]) groups according to the Evidence-based Clinical Practice Guideline for CKD 2018.^1^ A decrease in the renal function and increase in the incidence of CA-AKI were observed in proportion to the increase in the proteinuria.

ACEI, angiotensin-converting enzyme inhibitor; ARB, angiotensin II receptor blocker; BMI, body mass index; CA-AKI, contrast-associated acute kidney injury; CKD, chronic kidney disease; DBP, diastolic blood pressure; ECMO, extracorporeal membrane oxygenation; eGFR, estimated glomerular filtration rate; FBS, fasting blood sugar; HbA1c, hemoglobin A1c; HDL-C, high-density lipoprotein cholesterol; IABP, intra-aortic balloon pumping; LDL-C, low-density lipoprotein cholesterol; LVEF, left ventricular ejection fraction; MRA, mineralocorticoid receptor inhibitor; NT-proBNP, N-terminal pro-brain natriuretic peptide; NSTEMI, non-ST elevation myocardial infarction; SBP, systolic blood pressure; SCr, serum creatinine; STEMI, ST elevation myocardial infarction. Data are presented as the number, frequency, and mean ± SD. *, excluding metformin.

Reference

1.Evidence-based clinical practice guideline for CKD 2018 (in Japanese). *Japanese Society of Nephrology*. 2018.

**Supplementary Table 2. Baseline characteristics of the patients in the derivation and validation dataset.**

|  | **Derivation dataset**  **(n = 840)** | **Validation dataset**  **(n = 414)** | **P-value** |
| --- | --- | --- | --- |
| Age (years) | 70.1 ± 11.1 | 70.2 ± 11.1 | 0.97 |
| Male, n (%) | 597 (71.2) | 308 (74.4) | 0.22 |
| BMI (kg/m^2^) | 23.9 ± 4.1 | 23.8 ± 4.4 | 0.65 |
| SBP (mmHg) | 134 ± 22 | 132 ± 21 | 0.12 |
| DBP (mmHg) | 71 ± 22 | 70 ± 14 | 0.02 |
| Emergency procedure, n (%) | 147 (17.5) | 86 (20.8) | 0.17 |
| STEMI | 92 (11.0) | 53 (12.8) | 0.35 |
| NSTEMI | 55 (6.6) | 35 (8.5) | 0.24 |
| **Comorbidities, n (%)** |  |  |  |
| Hypertension | 666 (79.4) | 335 (80.9) | 0.52 |
| Diabetes mellitus | 343 (40.9) | 167 (40.3) | 0.85 |
| Heart failure | 181 (21.6) | 95 (22.9) | 0.58 |
| Dyslipidemia | 583 (69.5) | 275 (66.4) | 0.28 |
| Prior myocardial infarction | 257 (30.6) | 129 (31.2) | 0.85 |
| CKD | 371 (44.2) | 183 (44.2) | 1.00 |
| Smoking | 221 (26.3) | 110 (26.6) | 0.93 |
| **Laboratory data** |  |  |  |
| Hematocrit (%) |  |  |  |
| LDL-C (mg/dL) | 106.1 ± 35.7 | 107.6 ± 35.5 | 0.49 |
| HDL-C (mg/dL) | 50.2 ± 14.4 | 50.6 ± 14.3 | 0.66 |
| Triglyceride (mg/dL) | 153.3 ± 99.3 | 144.7 ± 85.1 | 0.12 |
| FBS (mg/dL) | 144.9 ± 60.2 | 146.5 ± 68.3 | 0.68 |
| HbA1c (%) | 6.7 ± 3.2 | 6.7 ± 3.0 | 0.81 |
| SCr (mg/dL) | 1.0 ± 0.4 | 1.0 ± 0.3 | 0.56 |
| eGFR (ml/min/1.73m^2^) | 61.9 ± 19.1 | 62.1 ± 20.2 | 0.87 |
| NT-proBNP (pg/mL) | 1175 ± 4219 | 1402 ± 4642 | 0.40 |
| Proteinuria, (n) (%) | 204 (24.3) | 103 (24.9) | 0.83 |
| LVEF (%) | 60.6 ± 12.5 | 61.7 ± 12.2 | 0.13 |
| Contrast media volume (ml) | 175.8 ± 62.0 | 176.4 ± 65.8 | 0.87 |
| IABP/ECMO, n (%) | 18 (2.1) | 14 (3.4) | 0.23 |
| **Medications**, n (%) |  |  |  |
| Aspirin | 699 (83.3) | 333 (80.4) | 0.22 |
| ACEI | 104 (12.4) | 51 (12.3) | 0.97 |
| ARB | 434 (51.7) | 214 (51.7) | 0.99 |
| β-blocker | 283 (33.7) | 140 (33.8) | 0.97 |
| Calcium channel blocker | 380 (45.3) | 201 (48.6) | 0.28 |
| Oral hypoglycemic agents* | 294 (35.0) | 150 (36.2) | 0.68 |
| Metformin | 80 (9.5) | 38 (9.2) | 0.84 |
| Insulin | 47 (5.6) | 22 (5.3) | 0.83 |
| Statin | 556 (66.3) | 272 (65.7) | 0.84 |
| Loop diuretics | 164 (19.5) | 77 (18.6) | 0.69 |
| MRA | 56 (6.7) | 16 (3.9) | 0.03 |
| CA-AKI, n (%) | 39 (4.6) | 25 (6.0) | 0.29 |
| In-hospital hemodialysis, n (%) | 8 (1.0) | 8 (1.9) | 0.20 |
| Chronic hemodialysis, n(%) | 9 (1.1) | 7 (1.7) | 0.40 |
| In-hospital mortality, n (%) | 12 (1.4) | 5 (1.2) | 0.74 |

Abbreviations were presented in Supplementary Table 1. *, excluding metformin. Data are presented as the number, frequency, and mean ± SD.

**Supplementary Table 3. Univariate logistic analysis**

| **Variables** | **OR (95% CI)** | **p-value** |
| --- | --- | --- |
| Age, 1 year | 1.04 (1.01-1.06) | <0.01 |
| Sex | 1.60 (0.95-2.70) | 0.08 |
| BMI, per 1 kg/m^2^ | 0.91 (0.85-0.97) | <0.01 |
| SBP, per 1 mmHg | 0.99 (0.98-1.01) | 0.35 |
| DBP, per 1 mmHg | 0.99 (0.97-1.01) | 0.34 |
| Emergency procedure | 3.74 (2.23-6.26) | <0.01 |
| STEMI | 3.27 (1.84-5.82) | <0.01 |
| NSTEMI | 2.57 (1.26-5.23) | 0.02 |
| Comorbidities |  |  |
| Hypertension | 3.09 (1.23-7.78) | <0.01 |
| Diabetes mellitus | 1.59 (0.96-2.62) | 0.07 |
| Heart failure | 5.06 (3.03-8.46) | <0.01 |
| Dyslipidemia | 0.50 (0.30-0.83) | <0.01 |
| Prior myocardial infarction | 0.61 (0.33-1.12) | 0.10 |
| CKD | 7.43 (3.75-14.7) | <0.01 |
| Smoking | 1.09 (0.62-1.91) | 0.76 |
| **Laboratory data** |  |  |
| Hematocrit, per 1% | 1.01 (0.99-1.03) | 0.67 |
| LDL-C, per 1 mg/dL | 1.00 (1.00-1.01) | 0.55 |
| HDL-C, per 1 mg/dL | 1.00 (0.98-1.02) | 0.91 |
| Triglyceride, per 1 mg/dL | 1.00 (1.00-1.00) | 0.66 |
| FBS, per 1 mg/dL | 1.00 (1.00-1.01) | 0.18 |
| HbA1c, per 1% | 1.01 (0.94-1.07) | 0.85 |
| SCr, per 1 mg/dL | 6.43 (3.64-11.4) | <0.01 |
| eGFR, per 1 mL/min/1.73m^2^ | 0.93 (0.92-0.95) | <0.01 |
| NT-proBNP, per 1 pg/mL | 1.00 (1.00-1.00) | <0.01 |
| LVEF, per 1% | 0.95 (0.94-0.97) | <0.01 |
| Contrast media volume, per 1mL | 1.00 (1.00-1.01) | 0.13 |
| Proteinuria ([-] vs. [+/-, +-3+]) | 6.16 (3.63-10.5) | <0.01 |
| IABP/ECMO | 6.94 (2.98-16.1) | <0.01 |
| **Medications** |  |  |
| Aspirin | 0.33 (0.19-0.56) | <0.01 |
| ACEI | 1.88 (0.05-1.00) | 0.06 |
| ARB | 0.93 (0.56-1.54) | 0.77 |
| β-blocker | 0.95 (0.56-1.63) | 0.86 |
| Calcium channel blocker | 1.74 (1.04-2.90) | 0.03 |
| Oral hypoglycemic agents* | 1.54 (0.93-2.56) | 0.10 |
| Metformin | 0.81 (0.32-2.05) | 0.64 |
| Insulin | 2.23 (0.98-5.10) | 0.06 |
| Statin | 0.33 (0.20-0.55) | <0.01 |
| Loop diuretics | 3.09 (1.83-5.19) | <0.01 |
| MRA | 1.42 (0.55-3.66) | 0.49 |

Abbreviations were presented in Supplementary Table 1. OR = odds ratio, CI = confidence interval. *, excluding metformin.

**Supplementary Figure 1.**

(A) Derivation dataset (B) Validation dataset


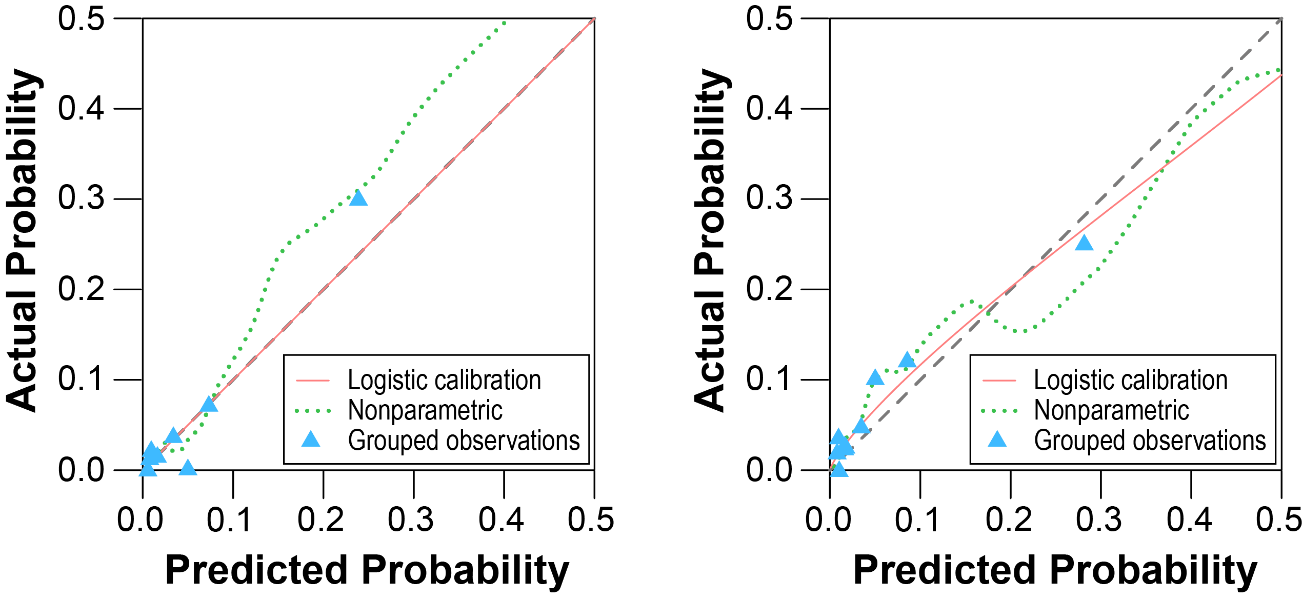


Calibration curves of the logistic regression model (apparent), nonparametric model (bias-corrected) and grouped observation (8 groups) are shown. The calibration curves of the logistic regression showed no relevant departures from the ideal predictions.

**Sample size estimation.**

We estimated the sample size using R project. We input “percentage of CA-AKI in patients undergoing PCI=0.05, odds ratio of our risk score of 0.21, alpha=0.05, and power=0.8,” and it revealed that 606 patients should be included. Our study included a sufficient number of patients.
